# If I had a Hammer

**DOI:** 10.1186/1749-8090-10-S1-A331

**Published:** 2015-12-16

**Authors:** M Tarazi, N Mayooran, S Shannon, J Hinchion

**Affiliations:** 1 Department of Cardiothoracic Surgery, Cork University Hospital, Cork, Ireland

## Background/Introduction

We present the case of a 64 year old man who was referred to our tertiary center with 3 nails drilled into his chest with a nail gun secondary to deliberate self-harm. The patient underwent a sternotomy for removal of the 3 nails. 2 nails were easily visible and removed. They caused pericardial perforation and myocardial injury. The third nail was difficult to locate. Myocardial injury showed a trajectory near the junction of the left anterior descending artery and the diagonal artery with no bleeding appreciated. A magnet was then used to locate the third nail which was found to be deep in the hilum of the lung. The magnet was then used to gently 'milk' the nail into a more superficial position. Once the nail was more easily accessible, it was removed.

## Discussion/Conclusion

A case of a lost metal during surgery can present a challenge to even the most experienced surgeons. Currently, the protocol involves a standard visual search followed by intra-operative imaging. This approach can lengthen operative time, cause injury during a rigorous search, and increase costs. Our case demonstrates how the use of magnets can assist a surgeon in locating and removing lost metal reducing the risk of iatrogenic injury, length of the operation and cost.

## Consent

Written informed consent was obtained from the patient for publication of this abstract and any accompanying images. A copy of the written consent is available for review by the Editor of this journal.

